# Comparing automated subcortical volume estimation methods; amygdala volumes estimated by FSL and FreeSurfer have poor consistency

**DOI:** 10.1002/hbm.70027

**Published:** 2024-11-26

**Authors:** Patrick Sadil, Martin A. Lindquist

**Affiliations:** ^1^ Department of Biostatistics Johns Hopkins Bloomberg School of Public Health Baltimore Maryland USA

**Keywords:** amygdala, MRI

## Abstract

Subcortical volumes are a promising source of biomarkers and features in biosignatures, and automated methods facilitate extracting them in large, phenotypically rich datasets. However, while extensive research has verified that the automated methods produce volumes that are similar to those generated by expert annotation; the consistency of methods with each other is understudied. Using data from the UK Biobank, we compare the estimates of subcortical volumes produced by two popular software suites: FSL and FreeSurfer. Although most subcortical volumes exhibit good to excellent consistency across the methods, the tools produce diverging estimates of amygdalar volume. Through simulation, we show that this poor consistency can lead to conflicting results, where one but not the other tool suggests statistical significance, or where both tools suggest a significant relationship but in opposite directions. Considering these issues, we discuss several ways in which care should be taken when reporting on relationships involving amygdalar volume.

## Graphical abstract

This article compares the estimates of subcortical volume provided by two popular software packages, FSL and FreeSurfer. Between the packages, there is good to excellent consistency for most subcortical regions, but the consistency of estimations of the volume of the amygdala is poor.

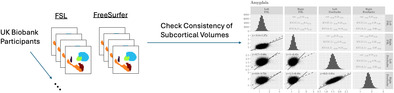

## 1 INTRODUCTION

Regional volumes of subcortex have been proposed as biomarkers for several psychopathologies. For example, the volume of the amygdala has been suggested as a biomarker for Alzheimer, depression symptom severity in young adults, bipolar disorder in youth, migraine frequency, chronic pain, and others (Daftary et al., 2019; Khatri & Kwon, 2022; Liu et al., 2017; Pfeifer et al., 2008; Rogers et al., 2009; Ruocco et al., 2012; Szeszko et al., 2004; Vachon‐Presseau et al., 2016). As a biomarker, subcortical volumes are advantageous for being interpretable (given the rich literature linking these structures to many functions), explainable (hypotrophy and hypertrophy are both easily described to healthcare providers and patients), and readily available. The latter point comes from the fact that it is possible to estimate the regional volumes from any structural image with several automated algorithms.

For estimating regional subcortical volumes, two automated techniques are popular: FMRIB's Integrated Registration and Segmentation Tool (FIRST) from the FMRIB Software Library (FSL) and FreeSurfer's Automated Segmentation (ASEG) (Fischl, 2012; Patenaude, 2007; Patenaude et al., 2011). Both techniques exhibit high consistency with the gold standard of manual segmentation in healthy adults and some clinical populations (Dewey et al., 2010; Doring et al., 2011; Hsu et al., 2002; Lehmann et al., 2010; Morey et al., 2009; Nugent et al., 2013; Pardoe et al., 2009; Tae et al., 2008; Wenger et al., 2014), although there is variability across regions and between methods. For segmenting the hippocampus, FreeSurfer has been reported as having higher intraclass correlations than FSL (Doring et al., 2011), and neither method appears to have worse reliability (across repeated scans) than manual segmentation (Mulder et al., 2014). For the putamen, FSL has a higher Dice coefficient with manual segmentation, and the methods perform similarly on the caudate (Perlaki et al., 2017). For the amygdala, which method performs better depends on the metric (Morey et al., 2009). However, these comparisons may not generalize to other populations (e.g., pediatric, elderly), given that performance of the automated techniques is not consistently high across populations (Schoemaker et al., 2016; Kim et al., 2012; Sánchez‐Benavides et al., 2010; Zhou et al., 2021). Both tools may remain popular because their performance against a gold standard depends on the research context, with different situations favoring different tools.

Given that both tools are often reasonable choices for a given study, and that the literature contains many reports that are based on one but not the other, we sought to understand how the tools compare with each other. To our knowledge, the two tools have only been compared with each other by Perlaki et al. (2017). In their research, the tools were consistent with each other (exhibiting intraclass correlations that ranged from around 0.7–0.9), but the analyses included only the putamen and caudate, and the sample size was relatively small (*N* = 30). That is, it remains unclear how often the two tools provide the same results and which factors affect these discrepancies.

We extend the results of Perlaki et al. (2017) to the remaining subcortical structures using a much larger population (tens of thousands). Our investigation should be considered in the context of research that uses estimates of volume as a biomarker by, for example, correlating it with a health‐related outcome. Our primary concern is whether the results of such a study are expected to depend on the method used for automated segmentation.

## 2 METHODS AND RESULTS

First, we looked at the consistency of subcortical volumes between FSL and FreeSurfer using data from the UK Biobank (Alfaro‐Almagro et al., 2018). The data were downloaded Jan 2024 and contained 45,743 participants with usable anatomical data (Category 190: FreeSurfer ASEG; Category 1102: FSL FIRST). Agreement and consistency of the tools were measured using the single‐measurement intraclass correlation coefficient. For details on intraclass correlation calculations, see Appendix A1. Estimates and associated uncertainty are displayed using subscripts, as recommended by Louis and Zeger (2008). For example, an estimate of 0.22 with a 95% confidence interval spanning [0.21, 0.23] will be rendered as 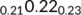.

Across all structures, estimated agreement was lower than estimated consistency (Table A1), reflecting differences in the average volumes estimated by the two methods (Appendix A2). However, a constant shift across participants would not affect many analyses targeted by our primary concern, analyses related to estimated correlations between regional volumes and some other measures. For that reason, we focus not on agreement but instead on consistency.

**FIGURE 1:**
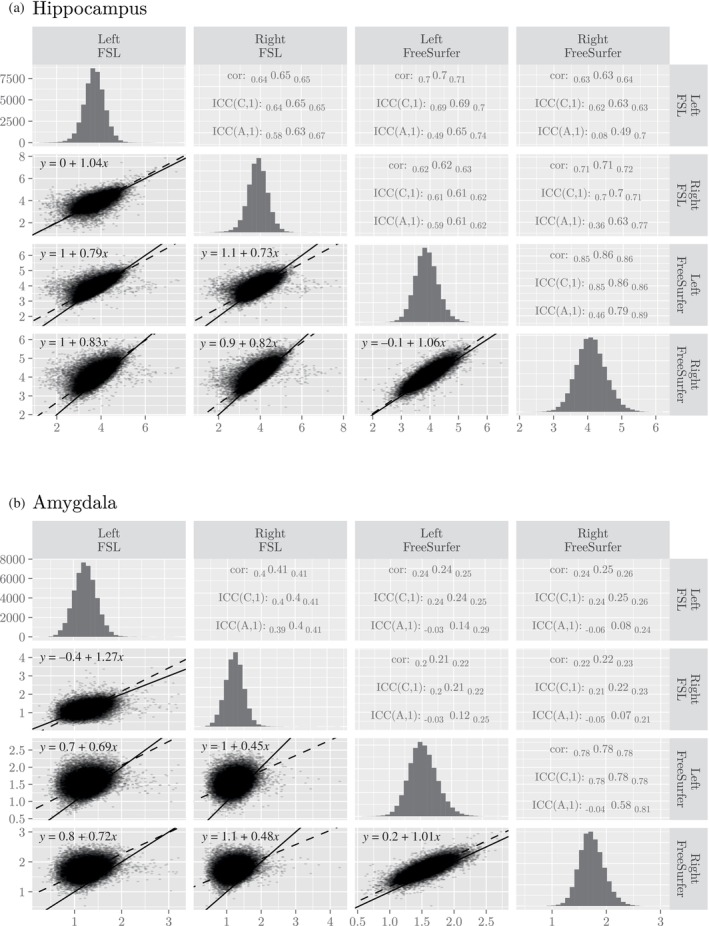
Comparisons of subcortical volumes estimated by FSL and FreeSurfer for two example regions (a) hippocampus and (b) amygdala. For the remaining subcortical regions, see Table A1. In the lower triangular panels, the line of equivalence is marked with a solid line, and the dashed lines show the result of orthogonal regression. In the upper panel, the uncertainty estimates span 95% confidence intervals. The histograms along the diagonal display volumes in the full sample.

Consistency varies by region (Figure 1, Table A1). To interpret the intraclass correlations, consider the categories provided by Cicchetti and Sparrow (1981): <0.4: poor, [0.4, 0.6): fair, [0.6, 0.75): good, [0.75, 1): excellent. Using those categories, the methods exhibit “good” to “excellent” consistency for most regions. However, the consistency of volumes for the amygdala is markedly worse than the others, being around only 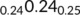 and 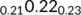 for the left and right hemispheres (ranges of uncertainty span 95% confidence intervals). Compare those values to the values for the hippocampus (Figure 1), which has good consistency (left: 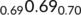, right: 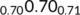). For both structures (i.e., the amygdala and hippocampus), consistency across hemispheres as reported by FreeSurfer is the highest numerically (Figure 1).

Although our focus is on the consistency between tools, we note that their lack of agreement may lead researchers to make conclusions about whether one hemisphere tends to have a larger amygdala than the other. There is an interaction between method and hemisphere (FSL–FreeSurfer: 0.22, *p* < .001), with FreeSurfer reporting that the right amygdala is larger than the left (left–right: −0.19, *p* < .001) and FSL reporting that the left is larger than the right (left–right: 0.04, *p* < .001). As the effect side is almost zero in the latter, practically the interaction is driven by the right side.

The amygdala has been described as particularly challenging to segment; one small study (*N* = 23) reports a consistency of 0.6 for volumes estimated by FSL across repeated scans of the same individual (Morey et al., 2010). Moreover, automated segmentation algorithms can be affected by experimental factors like site, scanner, participant positioning, and software version (Du et al., 2021; Hedges et al., 2022; Liu et al., 2020; McGuire et al., 2017; Morey et al., 2010; Mulder et al., 2014; Perlaki et al., 2017; Yang et al., 2016), and differential sensitivity to such factors could impact an intraclass correlation. In the UKB, several potentially confounding factors were correlated with estimates of amygdalar volume (Figure A2). However, regressing these factors from the estimates of volume did not improve the consistency between the methods (Appendix A4).

With poor consistency between measurements of the amygdala, there is concern that reported relationships involving amygdala volumes may depend on which method is used for estimating the volume, a choice that may be considered arbitrary or lab‐specific. At least two kinds of issues could arise. First, lower consistency could make it more likely that one but not both methods lead to significant correlations. Second, lower consistency could make it more likely that the two methods produce significant correlations that go in opposite directions.

To investigate how often these two issues could occur, we first simulated experiments with artificial data. In each simulation, datasets with two noisy estimates of volume and a third, outcome, variable were generated such that the two‐volume estimates had a pre‐specified intraclass correlation with each other (in expectation), and the true volume had a given product–moment correlation with the outcome variable. The estimated volumes were then tested for a product–moment correlation with the outcome, and the process was repeated for several intraclass correlations and sample sizes. In all simulations, the true product–moment correlation was set to a value that is either typical for neuroimaging research (0.1, Marek et al., 2022), small but non‐zero (0.01), or large (0.2). For additional details on the simulation methods, see Appendix A5.

**FIGURE 2:**
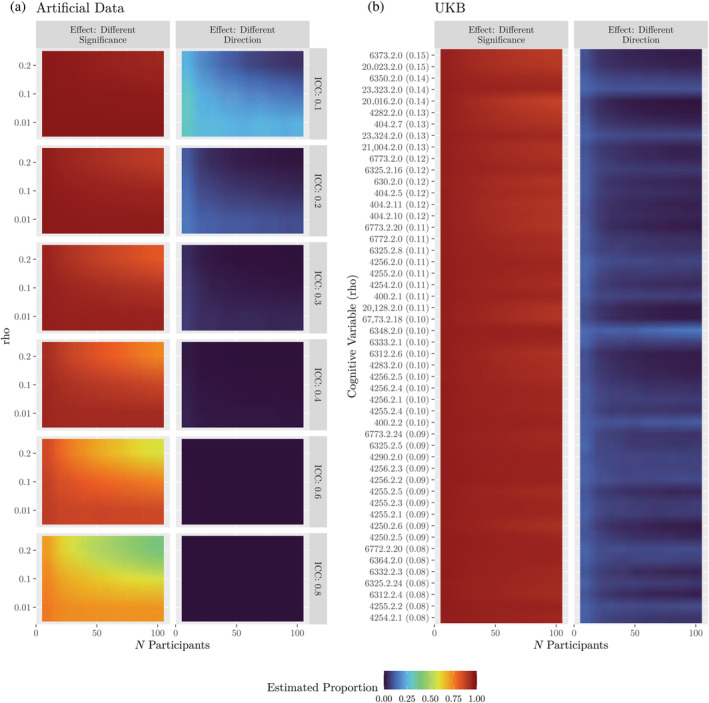
Effects of low measurement consistency. In the subfigures, the left column or panel shows the proportion of simulated experiments where the methods produce correlations on opposite sides of an α=0.05 significance threshold. The right column or panel shows the proportion of simulated experiments where both measures are significantly correlated with an outcome but in opposite directions. Proportions are shown with color (Swihart et al., 2010). (a) Simulations with artificial data. Rows (rho) indicate pre‐specified product–moment correlation with the outcome variable. ICC: Pre‐specified intraclass correlation between measures of volume. (b) Simulations with UKB. Each row corresponds to a non‐image derived phenotype from the UKB. The value in parentheses is the absolute correlation of the variable with the average volumes of the left amygdala (average across methods). For a display without color that includes estimates of uncertainty, see Figure A3.

With lower consistency, the estimated product–moment correlations were more often on opposite sides of a significance threshold (Figure 2a, left column). For example, with consistency near the value that was estimated for volumes of the amygdala in the UKB (0.2), a typical effect size (0.1), and experiments with 50 participants, significance differed in around 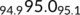 percent of simulations in which one test was significant (for simulations, estimates indicate medians and ranges of uncertainty span 95% equal‐tailed intervals, see Appendix A5). With a sample size of 100, that percentage was only minimally different (to 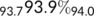). For a discussion on this insensitivity to sample size, see Section Appendix A.6.

Lower consistency also coincided with a higher proportion of experiments in which the two methods correlate in opposite directions (Figure 2a, right column). Considering the previous example (an intraclass correlation of 0.2, an effect size of 0.1, and 50 participants per experiment), around 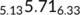 percent of experiments in which both product–moment correlations were significant resulted in the correlations having opposite signs. As the intraclass correlation increased, the rates of this effect decreased rapidly.

To assess how often these two issues could occur in practice, we repeated the above analyses but with the UKB. From the UKB, we extracted the “Cognitive” variables using the FMRIB UKBiobank Normalisation, Parsing and Cleaning Kit (McCarthy, 2023), restricting analyses to variables with instance 2 and that exhibited one of the largest (in absolute magnitude) 50 rank correlations (between the cognitive variables and the average of the two estimates of the volume of the left amygdala), and further to only those participants that had values for all 50 of those variables (23,486 participants). Each simulation resulted in a rank correlation between the two volume estimates for the left amygdala and each of the 50 variables (results were comparable with the right amygdala). For additional details, see Appendix A5.

Considering differing significance levels, the rates across experiments using the UKB resembled the rates from experiments with artificial data. In simulated experiments with 50 UKB participants in which at least one correlation was significant, one correlation was not significant in between 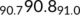 and 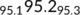 percent of simulations (the two estimates cover the 50 cognitive variables; Figure 2b, left panel). Proportions were generally lower for variables that were more strongly correlated with the measure of volume (for illustration, compare the variables that are higher versus lower in the left panel of Figure 2b).

Considering significant correlations with differing signs, the rates across experiments with the UKB bracketed the rates with artificial data. In experiments simulated with 50 UKB participants, the rates ranged from 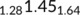 to 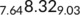. For most variables, increasing the number of participants decreased the proportion of experiments in which the two correlations exhibited opposite signs, but for some the proportion increased. Across the 50 variables, 9 had a higher proportion at *N* = 100 than *N* = 50, 2 of which had non‐overlapping 95% equal‐tailed intervals (6348: duration to complete numeric path; 400: time to complete round of pairs matching game; see also Figure A3).

## 3 DISCUSSION

We examined the consistency of subcortical volumes within the UK Biobank (Alfaro‐Almagro et al., 2018), observing that two common methods of estimating the volume of the amygdala, one from FSL and one from FreeSurfer, have poor consistency with each other.

We speculate that the lack of consistency for the amygdala compared with other subcortical structures is due in part to its relatively small size. For larger structures, discrepancies in a few voxels would have a smaller impact on consistency as compared with the impact of the same discrepancy on a smaller structure. When considering the difference in amygdala volume estimates between tools as a proportion of their average (Figure A1b), a trend is revealed where smaller structures tend to have more variable differences than larger ones. The amygdala is the smallest structure analyzed and has the largest variation. Even within other structures, the variability of the proportional difference increases as the average size decreases. Moreover, there is a clear correlation between head size and difference in amygdala volume (Figure A2), whereby smaller heads are associated with larger differences. Hence, smaller structures may be more difficult to segment consistently due to the proportional impact of discrepancies in the classification of a few voxels.

The main concern in this report is that consistency this poor can lead to conflicting results. Two kinds of conflict were explored: the methods producing correlations that are on opposite sides of significance thresholds, and the methods producing volumes with significant correlations that have differing signs. The prevalence of these occurrences was estimated with artificial data and data from the UKB. Based on the observed rates, we make the following recommendations.

### 3.1 Recommendations

#### 3.1.1 When testing for new biomarkers, report relationships with multiple automated methods (e.g., both FSL and FreeSurfer)

Researchers may have idiosyncratic reasons for selecting a method, particularly when the choice is viewed as arbitrary. If the choice between methods is arbitrary, then reporting the outcome across selections clarifies the fragility or robustness of a result (for a general discussion, see Steegen et al., 2016). The choice may not always be arbitrary, as there are metrics along which and study populations for which one method may perform better (Huizinga et al., 2021; Morey et al., 2009; Zhou et al., 2021). Note that one effect of low consistency could be a downward bias on the magnitude of estimated correlations (Appendix A7), and so there may be advantages to not only reporting but also combining the estimates across methods when predicting health‐related outcomes (e.g., by averaging, or including both as independent variables in predictive models). Reporting estimates from multiple reasonable tools will help move conclusions beyond “there exists a correlation with the volume of the amygdala as estimated by method M (version x)” to simply “there exists a correlation with the volume of the amygdala.”

#### 3.1.2 When reviewing or conducting meta‐analyses of relationships with amygdala volume, consider the method that was used to estimate volume

As mentioned in the Introduction, the amygdala has received substantial attention due to being predictive of an array of health‐related outcomes. As presented in this report, the volume that is estimated by one automated method may only weakly correspond to the volume estimated by another, and so it may be misleading to conduct a meta‐analysis without accounting for the algorithms used by the individual studies. In the UKB, there are differences in the strength of the correlations between the measures of volume and the cognitive variables; in nearly all of the variables considered in this report, the magnitude of the correlations involving FreeSurfer's method was numerically larger (Figure A5). Larger correlations do not imply higher veracity, but they further indicate that the methods track aspects of amygdalar anatomy differently.

#### 3.1.3 When replicating or extending research on a relationship that involves the volume of the amygdala, use the method reported in the original publications

This recommendation follows standard practice for a replication study. We highlight it here in consideration of both extension studies that aim to apply a biomarker or biosignature that includes amygdala volume (such as when testing a putative relationship in a new population), and also in consideration of the ongoing evolution of methods for automatically estimating subcortical volumes. Although FSL and FreeSurfer are two of the most popular methods, others exist (e.g., Akhondi‐Asl & Warfield, 2013), including newer techniques based on deep‐learning approaches (e.g., Billot et al., 2023; for review, see Singh & Singh, 2021). Newer methods may have better correspondence with manual segmentation, warranting their use in replication or extension studies. But as this report shows, two methods can perform well while exhibiting poor consistency with each other. So when building on prior findings, it remains important to use the methods of those prior findings, even when they are superseded.

## AUTHOR CONTRIBUTIONS

**Patrick Sadil**: Conceptualization; methodology; software; validation; formal analysis; investigation; resources; data curation; writing—original draft; writing—review and editing; visualization. **Martin A. Lindquist**: Conceptualization; methodology; validation; formal analysis; resources; writing—original draft; writing—review and editing; supervision; project administration; funding acquisition.

## CONFLICT OF INTEREST STATEMENT

The authors declare that they have no known competing financial interests or personal relationships that could have appeared to influence the work reported in this article.

## Supporting information


**Data S1.** Supporting Information.

## Data Availability

The data that support the findings of this study are available from UK Biobank. Restrictions apply to the availability of these data, which were used under license for this study. Data are available from https://www.ukbiobank.ac.uk/enable-your-research/apply-for-access with the permission of UK Biobank. Code to reproduce analyses is available on GitHub: https://github.com/psadil/auto-volume-comparisons.

## References

[hbm70027-bib-0001] Akhondi‐Asl, A. , & Warfield, S. K. (2013). Simultaneous truth and performance level estimation through fusion of probabilistic segmentations. IEEE Transactions on Medical Imaging, 32, 1840–1852. 10.1109/TMI.2013.2266258 23744673 PMC3788853

[hbm70027-bib-0002] Alfaro‐Almagro, F. , Jenkinson, M. , Bangerter, N. K. , Andersson, J. L. , Griffanti, L. , Douaud, G. , Sotiropoulos, S. N. , Jbabdi, S. , Hernandez‐Fernandez, M. , Vallee, E. , Vidaurre, D. , Webster, M. , McCarthy, P. , Rorden, C. , Daducci, A. , Alexander, D. C. , Zhang, H. , Dragonu, I. , Matthews, P. M. , … Smith, S. M. (2018). Image processing and quality control for the first 10,000 brain imaging datasets from UK biobank. NeuroImage, 166, 400–424. 10.1016/j.neuroimage.2017.10.034 29079522 PMC5770339

[hbm70027-bib-0003] Billot, B. , Greve, D. N. , Puonti, O. , Thielscher, A. , van Leemput, K. , Fischl, B. , Dalca, A. V. , & Iglesias, J. E. (2023). Synthseg: Segmentation of brain MRI scans of any contrast and resolution without retraining. Medical Image Analysis, 86, 102789. 10.1016/j.media.2023.102789 36857946 PMC10154424

[hbm70027-bib-0004] Cicchetti, D. V. , & Sparrow, S. A. (1981). Developing criteria for establishing interrater reliability of specific items: Applications to assessment of adaptive behavior. American Journal of Mental Deficiency, 86, 127–137. 7315877

[hbm70027-bib-0005] Daftary, S. , van Enkevort, E. , Kulikova, A. , Legacy, M. , & Brown, E. S. (2019). Relationship between depressive symptom severity and amygdala volume in a large community‐based sample. Psychiatry Research: Neuroimaging, 283, 77–82. 10.1016/j.pscychresns.2018.12.005 30554129

[hbm70027-bib-0006] Dewey, J. , Hana, G. , Russell, T. , Price, J. , McCaffrey, D. , Harezlak, J. , Sem, E. , Anyanwu, J. C. , Guttmann, C. R. , Navia, B. , Cohen, R. , & Tate, D. F. (2010). Reliability and validity of MRI‐based automated volumetry software relative to auto‐assisted manual measurement of subcortical structures in HIV‐infected patients from a multisite study. NeuroImage, 51, 1334–1344. 10.1016/j.neuroimage.2010.03.033 20338250 PMC2884380

[hbm70027-bib-0007] Doring, T. M. , Kubo, T. T. , Cruz, L. C. H. , Juruena, M. F. , Fainberg, J. , Domingues, R. C. , & Gasparetto, E. L. (2011). Evaluation of hippocampal volume based on MR imaging in patients with bipolar affective disorder applying manual and automatic segmentation techniques. Journal of Magnetic Resonance Imaging, 33, 565–572. 10.1002/jmri.22473 21563239

[hbm70027-bib-0008] Du, J. , Liang, P. , He, H. , Tong, Q. , Gong, T. , Qian, T. , Sun, Y. , Zhong, J. , & Li, K. (2021). Reproducibility of volume and asymmetry measurements of hippocampus, amygdala, and entorhinal cortex on traveling volunteers: A multisite mp2rage prospective study. Acta Radiologica, 62, 1381–1390. 10.1177/0284185120963919 33121264

[hbm70027-bib-0009] Fischl, B. (2012). Freesurfer. NeuroImage, 62, 774–781. 10.1016/j.neuroimage.2012.01.021 22248573 PMC3685476

[hbm70027-bib-0010] Hedges, E. P. , Dimitrov, M. , Zahid, U. , Brito Vega, B. , Si, S. , Dickson, H. , McGuire, P. , Williams, S. , Barker, G. J. , & Kempton, M. J. (2022). Reliability of structural MRI measurements: The effects of scan session, head tilt, inter‐scan interval, acquisition sequence, freesurfer version and processing stream. NeuroImage, 246, 118751. 10.1016/j.neuroimage.2021.118751 34848299 PMC8784825

[hbm70027-bib-0011] Hsu, Y. , Schuff, N. , Du, A. , Mark, K. , Zhu, X. , Hardin, D. , & Weiner, M. W. (2002). Comparison of automated and manual MRI volumetry of hippocampus in normal aging and dementia. Journal of Magnetic Resonance Imaging, 16, 305–310. 10.1002/jmri.10163 12205587 PMC1851676

[hbm70027-bib-0012] Huizinga, W. , Poot, D. H. J. , Vinke, E. J. , Wenzel, F. , Bron, E. E. , Toussaint, N. , Ledig, C. , Vrooman, H. , Ikram, M. A. , Niessen, W. J. , Vernooij, M. W. , & Klein, S. (2021). Differences between MR brain region segmentation methods: Impact on single‐subject analysis. Frontiers in Big Data, 4, 577164. 10.3389/fdata.2021.577164 34723175 PMC8552517

[hbm70027-bib-0013] Khatri, U. , & Kwon, G. R. (2022). Alzheimer's disease diagnosis and biomarker analysis using resting‐state functional MRI functional brain network with multi‐measures features and hippocampal subfield and amygdala volume of structural mri. Frontiers in Aging Neuroscience, 14, 818871. 10.3389/fnagi.2022.818871 35707703 PMC9190953

[hbm70027-bib-0014] Kim, H. , Chupin, M. , Colliot, O. , Bernhardt, B. C. , Bernasconi, N. , & Bernasconi, A. (2012). Automatic hippocampal segmentation in temporal lobe epilepsy: Impact of developmental abnormalities. NeuroImage, 59, 3178–3186. 10.1016/j.neuroimage.2011.11.040 22155377

[hbm70027-bib-0015] Lehmann, M. , Douiri, A. , Kim, L. G. , Modat, M. , Chan, D. , Ourselin, S. , Barnes, J. , & Fox, N. C. (2010). Atrophy patterns in Alzheimer's disease and semantic dementia: A comparison of freesurfer and manual volumetric measurements. NeuroImage, 49, 2264–2274. 10.1016/j.neuroimage.2009.10.056 19874902

[hbm70027-bib-0016] Liu, H. Y. , Chou, K. H. , Lee, P. L. , Fuh, J. L. , Niddam, D. M. , Lai, K. L. , Hsiao, F. J. , Lin, Y. Y. , Chen, W. T. , Wang, S. J. , & Lin, C. P. (2017). Hippocampus and amygdala volume in relation to migraine frequency and prognosis. Cephalalgia, 37, 1329–1336. 10.1177/0333102416678624 27919022

[hbm70027-bib-0017] Liu, S. , Hou, B. , Zhang, Y. , Lin, T. , Fan, X. , You, H. , & Feng, F. (2020). Inter‐scanner reproducibility of brain volumetry: Influence of automated brain segmentation software. BMC Neuroscience, 21, 35. 10.1186/s12868-020-00585-1 32887546 PMC7472704

[hbm70027-bib-0018] Louis, T. A. , & Zeger, S. L. (2008). Effective communication of standard errors and confidence intervals. Biostatistics, 10, 1–2. 10.1093/biostatistics/kxn014 18550565 PMC2639348

[hbm70027-bib-0019] Marek, S. , Tervo‐Clemmens, B. , Calabro, F. J. , Montez, D. F. , Kay, B. P. , Hatoum, A. S. , Donohue, M. R. , Foran, W. , Miller, R. L. , Hendrickson, T. J. , Malone, S. M. , Kandala, S. , Feczko, E. , Miranda‐Dominguez, O. , Graham, A. M. , Earl, E. A. , Perrone, A. J. , Cordova, M. , Doyle, O. , … Dosenbach, N. U. F. (2022). Reproducible brain‐wide association studies require thousands of individuals. Nature, 603, 654–660. 10.1038/s41586-022-04492-9 35296861 PMC8991999

[hbm70027-bib-0020] McCarthy, P. (2023). funpack. Zenodo. 10.5281/ZENODO.1997626

[hbm70027-bib-0021] McGuire, S. A. , Wijtenburg, S. A. , Sherman, P. M. , Rowland, L. M. , Ryan, M. , Sladky, J. H. , & Kochunov, P. V. (2017). Reproducibility of quantitative structural and physiological MRI measurements. Brain and Behavior, 7, e00759. 10.1002/brb3.759 28948069 PMC5607538

[hbm70027-bib-0022] Morey, R. A. , Petty, C. M. , Xu, Y. , Pannu Hayes, J. , Wagner, H. R. , Lewis, D. V. , LaBar, K. S. , Styner, M. , & McCarthy, G. (2009). A comparison of automated segmentation and manual tracing for quantifying hippocampal and amygdala volumes. NeuroImage, 45, 855–866. 10.1016/j.neuroimage.2008.12.033 19162198 PMC2714773

[hbm70027-bib-0023] Morey, R. A. , Selgrade, E. S. , Wagner, H. R. , Huettel, S. A. , Wang, L. , & McCarthy, G. (2010). Scan–rescan reliability of subcortical brain volumes derived from automated segmentation. Human Brain Mapping, 31, 1751–1762. 10.1002/hbm.20973 20162602 PMC3782252

[hbm70027-bib-0024] Mulder, E. R. , de Jong, R. A. , Knol, D. L. , van Schijndel, R. A. , Cover, K. S. , Visser, P. J. , Barkhof, F. , & Vrenken, H. (2014). Hippocampal volume change measurement: Quantitative assessment of the reproducibility of expert manual outlining and the automated methods FreeSurfer and FIRST. NeuroImage, 92, 169–181. 10.1016/j.neuroimage.2014.01.058 24521851

[hbm70027-bib-0025] Nugent, A. C. , Luckenbaugh, D. A. , Wood, S. E. , Bogers, W. , Zarate, C. A. , & Drevets, W. C. (2013). Automated subcortical segmentation using FIRST: Test‐retest reliability, interscanner reliability, and comparison to manual segmentation: Reliability of automated segmentation using FIRST. Human Brain Mapping, 34, 2313–2329. 10.1002/hbm.22068 22815187 PMC3479333

[hbm70027-bib-0026] Pardoe, H. R. , Pell, G. S. , Abbott, D. F. , & Jackson, G. D. (2009). Hippocampal volume assessment in temporal lobe epilepsy: How good is automated segmentation? Epilepsia, 50, 2586–2592. 10.1111/j.1528-1167.2009.02243.x 19682030 PMC3053147

[hbm70027-bib-0027] Patenaude, B. (2007). Bayesian statistical models of shape and appearance for subcortical brain segmentation (PhD thesis). University of Oxford. 10.1016/j.neuroimage.2011.02.046 PMC3417233 21352927

[hbm70027-bib-0028] Patenaude, B. , Smith, S. M. , Kennedy, D. N. , & Jenkinson, M. (2011). A Bayesian model of shape and appearance for subcortical brain segmentation. NeuroImage, 56, 907–922. 10.1016/j.neuroimage.2011.02.046 21352927 PMC3417233

[hbm70027-bib-0029] Perlaki, G. , Horvath, R. , Nagy, S. A. , Bogner, P. , Doczi, T. , Janszky, J. , & Orsi, G. (2017). Comparison of accuracy between fsl's first and freesurfer for caudate nucleus and putamen segmentation. Scientific Reports, 7, 2418. 10.1038/s41598-017-02584-5 28546533 PMC5445091

[hbm70027-bib-0030] Pfeifer, J. C. , Welge, J. , Strakowski, S. M. , Adler, C. , & Delbello, M. P. (2008). Meta‐analysis of amygdala volumes in children and adolescents with bipolar disorder. Journal of the American Academy of Child & Adolescent Psychiatry, 47, 1289–1298. 10.1097/CHI.0b013e318185d299 18827720

[hbm70027-bib-0031] Rogers, M. A. , Yamasue, H. , Abe, O. , Yamada, H. , Ohtani, T. , Iwanami, A. , Aoki, S. , Kato, N. , & Kasai, K. (2009). Smaller amygdala volume and reduced anterior cingulate gray matter density associated with history of post‐traumatic stress disorder. Psychiatry Research: Neuroimaging, 174, 210–216. 10.1016/j.pscychresns.2009.06.001 19914045

[hbm70027-bib-0032] Ruocco, A. C. , Amirthavasagam, S. , & Zakzanis, K. K. (2012). Amygdala and hippocampal volume reductions as candidate endophenotypes for borderline personality disorder: A meta‐analysis of magnetic resonance imaging studies. Psychiatry Research: Neuroimaging, 201, 245–252. 10.1016/j.pscychresns.2012.02.012 22507760

[hbm70027-bib-0050] Sánchez‐Benavides, G., Gómez‐Ansón, B., Sainz, A., Vives, Y., Delfino, M., & Peña‐Casanova, J. (2010). Manual validation of freesurfer's automated hippocampal segmentation in normal aging, mild cognitive impairment, and alzheimer disease subjects. Psychiatry Research: Neuroimaging, 181, 219–225. 10.1016/j.pscychresns.2009.10.011 20153146

[hbm70027-bib-0033] Schoemaker, D. , Buss, C. , Head, K. , Sandman, C. A. , Davis, E. P. , Chakravarty, M. M. , Gauthier, S. , & Pruessner, J. C. (2016). Hippocampus and amygdala volumes from magnetic resonance images in children: Assessing accuracy of freesurfer and fsl against manual segmentation. NeuroImage, 129, 1–14. 10.1016/j.neuroimage.2016.01.038 26824403 PMC7243960

[hbm70027-bib-0034] Singh, M. K. , & Singh, K. K. (2021). A review of publicly available automatic brain segmentation methodologies, machine learning models, recent advancements, and their comparison. Annals of Neurosciences, 28, 82–93. 10.1177/0972753121990175 34733059 PMC8558983

[hbm70027-bib-0035] Steegen, S. , Tuerlinckx, F. , Gelman, A. , & Vanpaemel, W. (2016). Increasing transparency through a multiverse analysis. Perspectives on Psychological Science, 11, 702–712. 10.1177/1745691616658637 27694465

[hbm70027-bib-0036] Swihart, B. J. , Caffo, B. , James, B. D. , Strand, M. , Schwartz, B. S. , & Punjabi, N. M. (2010). Lasagna plots: A saucy alternative to spaghetti plots. Epidemiology, 21, 621–625. 10.1097/EDE.0b013e3181e5b06a 20699681 PMC2937254

[hbm70027-bib-0037] Szeszko, P. R. , MacMillan, S. , McMeniman, M. , Lorch, E. , Madden, R. , Ivey, J. , Banerjee, S. P. , Moore, G. J. , & Rosenberg, D. R. (2004). Amygdala volume reductions in pediatric patients with obsessive–compulsive disorder treated with paroxetine: Preliminary findings. Neuropsychopharmacology, 29, 826–832. 10.1038/sj.npp.1300399 14970831

[hbm70027-bib-0038] Tae, W. S. , Kim, S. S. , Lee, K. U. , Nam, E. C. , & Kim, K. W. (2008). Validation of hippocampal volumes measured using a manual method and two automated methods (freesurfer and ibaspm) in chronic major depressive disorder. Neuroradiology, 50, 569–581. 10.1007/s00234-008-0383-9 18414838

[hbm70027-bib-0039] Vachon‐Presseau, E. , Tétreault, P. , Petre, B. , Huang, L. , Berger, S. E. , Torbey, S. , Baria, A. T. , Mansour, A. R. , Hashmi, J. A. , Griffith, J. W. , Comasco, E. , Schnitzer, T. J. , Baliki, M. N. , & Apkarian, A. V. (2016). Corticolimbic anatomical characteristics predetermine risk for chronic pain. Brain, 139, 1958–1970. 10.1093/brain/aww100 27190016 PMC4939699

[hbm70027-bib-0040] Wenger, E. , Mårtensson, J. , Noack, H. , Bodammer, N. C. , Kühn, S. , Schaefer, S. , Heinze, H. J. , Düzel, E. , Bäckman, L. , Lindenberger, U. , & Lövdén, M. (2014). Comparing manual and automatic segmentation of hippocampal volumes: Reliability and validity issues in younger and older brains. Human Brain Mapping, 35, 4236–4248. 10.1002/hbm.22473 24532539 PMC6869097

[hbm70027-bib-0041] Yang, C. Y. , Liu, H. M. , Chen, S. K. , Chen, Y. F. , Lee, C. W. , & Yeh, L. R. (2016). Reproducibility of brain morphometry from short‐term repeat clinical mri examinations: A retrospective study. PLoS One, 11, e0146913. 10.1371/journal.pone.0146913 26812647 PMC4727912

[hbm70027-bib-0042] Zhou, Q. , Liu, S. , Jiang, C. , He, Y. , & Zuo, X. N. (2021). Charting the human amygdala development across childhood and adolescence: Manual and automatic segmentation. Developmental Cognitive Neuroscience, 52, 101028. 10.1016/j.dcn.2021.101028 34749182 PMC8578043

