# Supplementary material for: Comparing automated subcortical volume estimation methods; amygdala volumes estimated by FSL and FreeSurfer have poor consistency
Source: Hum Brain Mapp. 2024 Nov 26;45(17):e70027. doi: 10.1002/hbm.70027 (PMC11599616; doi:10.1002/hbm.70027)
Supplement: Supplementary file 1 — Data S1. Supporting Information. [file HBM-45-e70027-s001.pdf]

Appendix A. Supplementary Materials

Appendix A.1. Intraclass Correlation

The intraclass correlation was based on a two-way mixed linear model (McGraw and Wong, 1996). In the model, the volume x for the region of participant i as measured by method k was treated as equal to the sum of an intercept, ν, the "true" volume, λ_i, a method bias, c_k, and an error term, ϵ_{ik}

$$
\begin{aligned}
x_{ik} &= \nu + \lambda_i + c_k + \epsilon_{ik} \\
\sum_k c_k &= 0 \\
\lambda_i &\sim N(0, \sigma^2_\lambda) \\
\epsilon_{ik} &\sim N(0, \sigma^2_\epsilon)
\end{aligned}
$$

Note that the c_k terms are assumed fixed, with variance given by $\sigma^2_c = \sum_k c^2_k/(k-1)$.

An important assumption of this model is that the two methods are expected to have the same mean-squared error. The model does not include features that would allow for the errors in the two methods to be correlated (e.g., participants are not distinguished by characteristics that coincide with the methods performing better or worse).

The consistency version of the intraclass correlation, $ICC(C,1)$, and the absolute agreement, $ICC(A,1)$ were given as fractions of the variance components

$$
\begin{aligned}
ICC(C,1) &= \frac{\sigma^2_\lambda}{\sigma^2_\lambda + \sigma^2_\epsilon} \\
ICC(A,1) &= \frac{\sigma^2_\lambda}{\sigma^2_\lambda + \sigma^2_\epsilon + \sigma^2_c}
\end{aligned}
$$

Variance components and associated confidence intervals estimated using the `R` package `irr` (R Core Team, 2023; Gamer et al., 2019), which uses the mean square approach described by McGraw and Wong (1996).

Appendix A.2. Differences in Average Volumes

Although we primarily focused on the consistency of the methods, we also observed shifts in the average volumes reported by the two methods (Figure A1). Differences in averages across methods have been reported previously (Gomez-Ramirez et al., 2022; Perlaki et al., 2017; Dewey et al., 2010; Huizinga et al., 2021). FSL tends to report volumes that are larger than those from FreeSurfer for the Accumbens, Amygdala, Hippocampus, and Pallidum, whereas for the Caudate, Putamen, and Thalamus FSL tends to report values that are smaller than those from FreeSurfer (all $p < 0.0001$ for two-sided t-test). Across structures, the variability in the difference appears to covary with the average of the two volume estimates, increasing as the average volume estimate decreases (Figure A1 b).

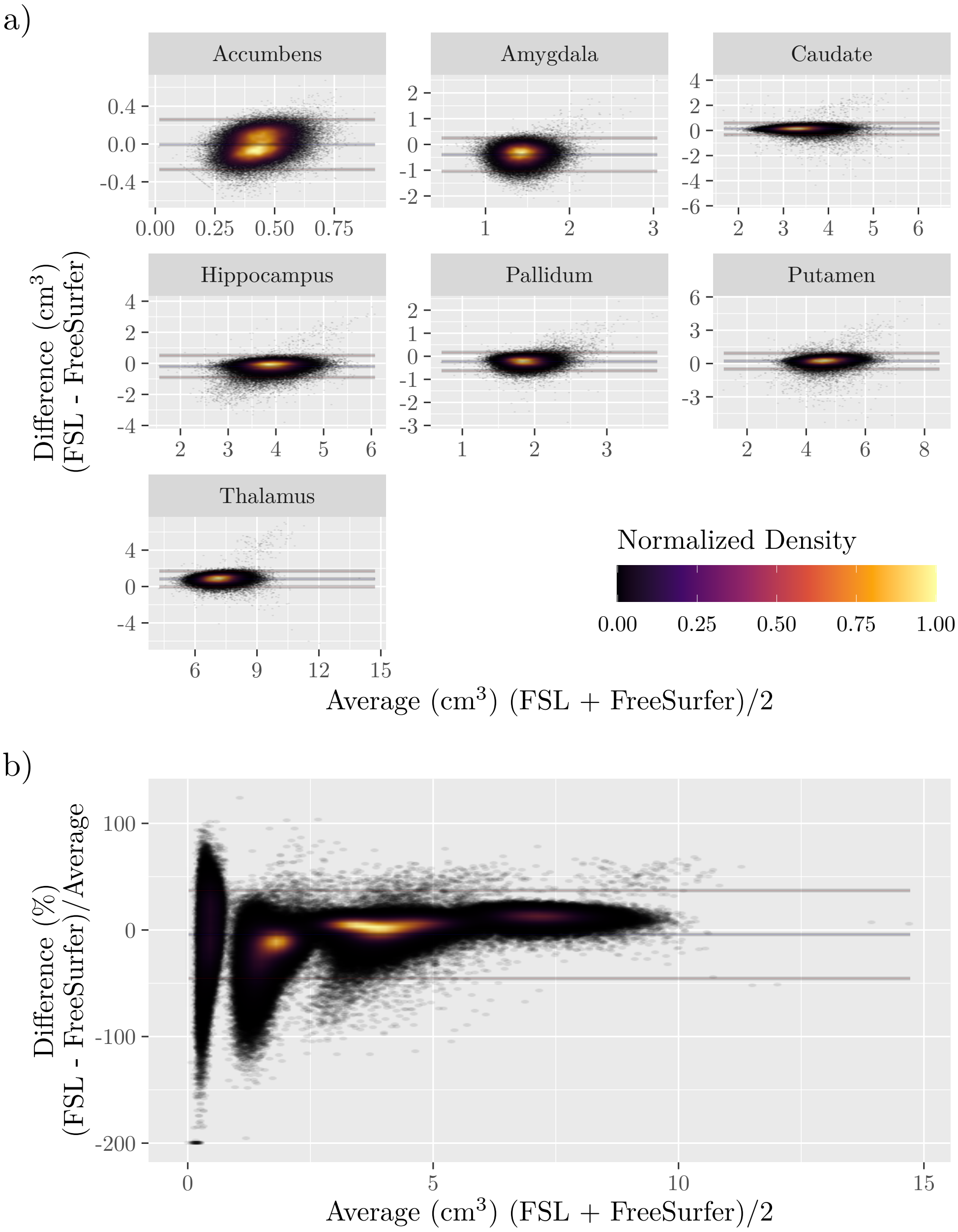

Figure A1: Bland-Altman Plots for Subcortical Volume. Horizontal lines show average difference and limits of agreement (1.96 standard deviations), with ribbons marking 95% confidence intervals. Panels correspond to subcortical structures. Left and right structures are plotted together. Shifts in the average estimate correspond to the central ribbon excluding zero. The color overlay indicates the degree of overplotting. a) Raw Differences. Note that axes are across panels independently. b) Differences by Percent Average.

Appendix A.3. Intraclass Correlations Across All Subcortical Regions

Structure	Hemisphere	ICC(C,1)	ICC(A,1)
Accumbens	Left	${}_{0.57}0.58_{0.58}$	${}_{0.05}0.44_{0.65}$
Accumbens	Right	${}_{0.56}0.57_{0.57}$	${}_{-0.03}0.38_{0.63}$
Amygdala	Left	${}_{0.24}0.24_{0.25}$	${}_{-0.03}0.14_{0.29}$
Amygdala	Right	${}_{0.21}0.22_{0.23}$	${}_{-0.05}0.07_{0.21}$
Caudate	Left	${}_{0.85}0.85_{0.86}$	${}_{0.75}0.83_{0.88}$
Caudate	Right	${}_{0.86}0.86_{0.87}$	${}_{0.62}0.82_{0.90}$
Hippocampus	Left	${}_{0.69}0.69_{0.70}$	${}_{0.49}0.65_{0.74}$
Hippocampus	Right	${}_{0.70}0.70_{0.71}$	${}_{0.36}0.63_{0.77}$
Pallidum	Left	${}_{0.68}0.68_{0.69}$	${}_{-0.09}0.41_{0.71}$
Pallidum	Right	${}_{0.66}0.67_{0.67}$	${}_{0.04}0.51_{0.73}$
Putamen	Left	${}_{0.79}0.79_{0.80}$	${}_{0.54}0.74_{0.84}$
Putamen	Right	${}_{0.82}0.83_{0.83}$	${}_{0.56}0.77_{0.87}$
Thalamus	Left	${}_{0.82}0.82_{0.83}$	${}_{-0.08}0.49_{0.79}$
Thalamus	Right	${}_{0.83}0.83_{0.83}$	${}_{-0.09}0.51_{0.81}$

Table A1: Intraclass Correlation for Subcortical Structures. Subscripts indicate 95% confidence intervals.

Appendix A.4. Intraclass Correlations After Residualizing on Potential Confounds

One possible source of low intraclass correlations could be systematic inaccuracies with certain kinds of participants. For example, one of the two methods could tend to underestimate volumes when given brains that have experienced severe atrophy, which would decrease the agreement of the methods. To assess this, correlations between the amygdala volumes and the "simple" confounds within the UKB were calculated (Alfaro-Almagro et al., 2021).

Several of the correlations appeared to be non-zero (Figure A2). However, regressing these confounds from the estimated amygdala volumes did not improve the intraclass correlations (Table A2).

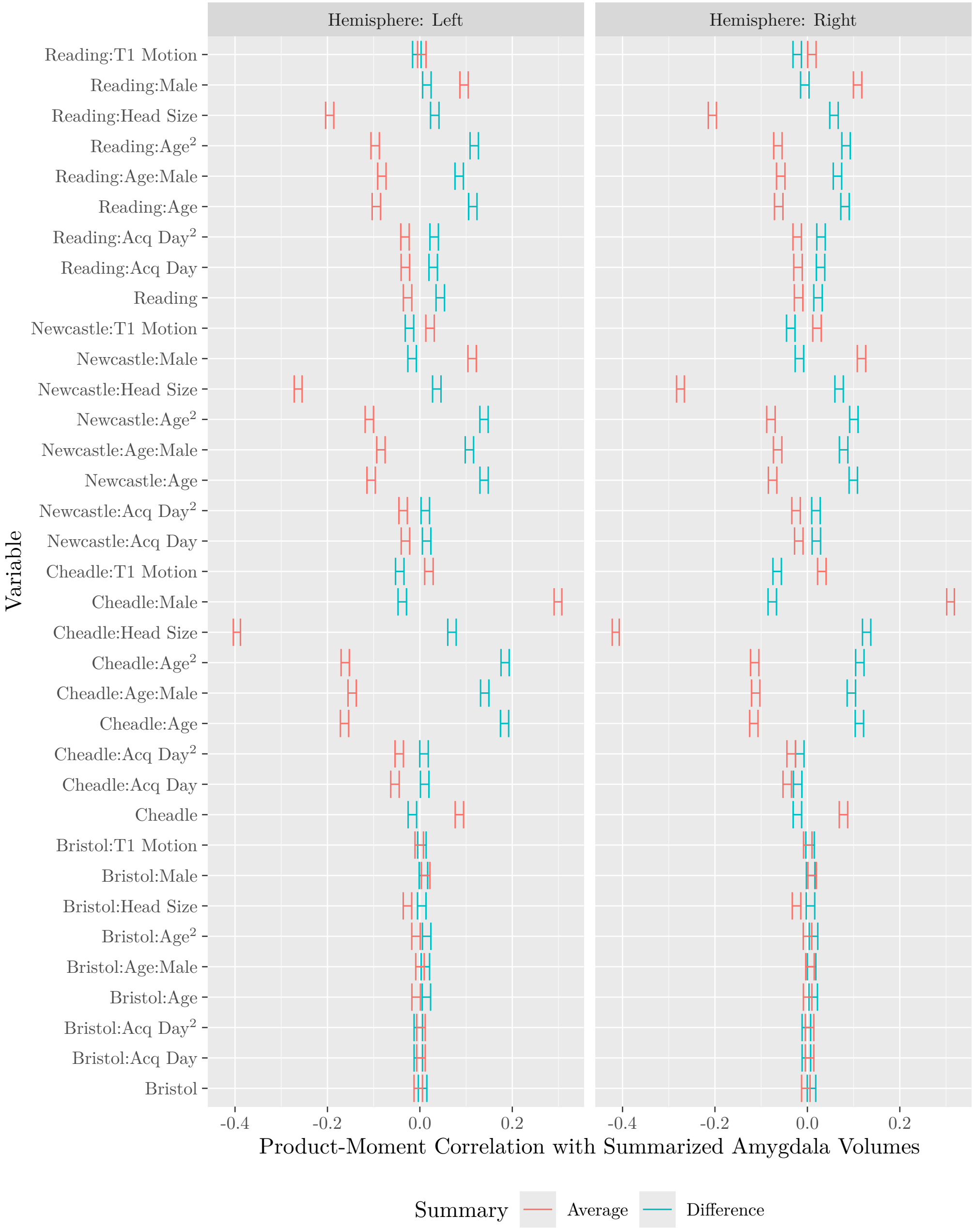

Figure A2: Correlation Between Potential Confounders and Amygdala Volume Measurements. Summary describes how the the volumes were combined across methods. Summaries were either an average or a difference (FSL-FreeSurfer). Note that the variable "Head Size" corresponds to a scaling factor, and that larger values imply smaller brains.

Structure	Hemisphere	ICC(C,1)	ICC(A,1)
Accumbens	Left	${}_{0.42}0.43_{0.44}$	${}_{0.01}0.30_{0.51}$
Accumbens	Right	${}_{0.44}0.45_{0.45}$	${}_{-0.05}0.27_{0.51}$
Amygdala	Left	${}_{0.07}0.08_{0.09}$	${}_{-0.01}0.04_{0.10}$
Amygdala	Right	${}_{0.04}0.05_{0.06}$	${}_{-0.01}0.01_{0.04}$
Caudate	Left	${}_{0.81}0.81_{0.81}$	${}_{0.68}0.78_{0.84}$
Caudate	Right	${}_{0.82}0.82_{0.82}$	${}_{0.52}0.76_{0.86}$
Hippocampus	Left	${}_{0.57}0.58_{0.59}$	${}_{0.36}0.53_{0.64}$
Hippocampus	Right	${}_{0.58}0.59_{0.59}$	${}_{0.23}0.50_{0.66}$
Pallidum	Left	${}_{0.55}0.56_{0.57}$	${}_{-0.09}0.30_{0.59}$
Pallidum	Right	${}_{0.54}0.54_{0.55}$	${}_{-0.01}0.38_{0.61}$
Putamen	Left	${}_{0.68}0.69_{0.69}$	${}_{0.38}0.62_{0.75}$
Putamen	Right	${}_{0.73}0.74_{0.74}$	${}_{0.41}0.66_{0.79}$
Thalamus	Left	${}_{0.64}0.65_{0.65}$	${}_{-0.08}0.27_{0.60}$
Thalamus	Right	${}_{0.64}0.65_{0.65}$	${}_{-0.09}0.28_{0.60}$

Table A2: Intraclass Correlation After Deconfounding. Prior to calculating consistency, volumes were deconfounded by a version of the "simple" parameter set described by Alfaro-Almagro et al. (2021). For the list of variables, see Figure A2. Subscripts indicate 95% confidence intervals.

Appendix A.4.1. Adjusting for ICV

When reporting differences in volume between groups, it is common to adjust for either head size or cerebral volume (Barnes et al., 2010; Mathalon et al., 1993; Voevodskaya et al., 2014). Adjusting by intracranial volume as estimated by FreeSurfer did not improve the intraclass correlations (Table A3).

Structure	Hemisphere	ICC(C,1)	ICC(A,1)
Accumbens	Left	$_{0.88}0.88_{0.88}$	$_{0.40}0.81_{0.91}$
Accumbens	Right	$_{0.90}0.90_{0.90}$	$_{0.23}0.81_{0.92}$
Amygdala	Left	$_{0.27}0.28_{0.29}$	$_{-0.03}0.17_{0.34}$
Amygdala	Right	$_{0.20}0.21_{0.22}$	$_{-0.05}0.07_{0.20}$
Caudate	Left	$_{0.79}0.80_{0.80}$	$_{0.67}0.77_{0.83}$
Caudate	Right	$_{0.81}0.81_{0.81}$	$_{0.52}0.75_{0.85}$
Hippocampus	Left	$_{0.63}0.63_{0.64}$	$_{0.42}0.58_{0.69}$
Hippocampus	Right	$_{0.63}0.64_{0.64}$	$_{0.28}0.55_{0.71}$
Pallidum	Left	$_{0.63}0.63_{0.64}$	$_{-0.09}0.36_{0.66}$
Pallidum	Right	$_{0.60}0.61_{0.61}$	$_{0.02}0.45_{0.68}$
Putamen	Left	$_{0.72}0.72_{0.72}$	$_{0.44}0.66_{0.78}$
Putamen	Right	$_{0.76}0.76_{0.76}$	$_{0.46}0.70_{0.81}$
Thalamus	Left	$_{0.75}0.75_{0.76}$	$_{-0.09}0.39_{0.72}$
Thalamus	Right	$_{0.75}0.76_{0.76}$	$_{-0.09}0.40_{0.73}$

Table A3: Consistency of Volumes when Adjusting by Intracranial Volume. Adjustments were done by residualizing with respect to ICV. Subscripts indicate 95% confidence intervals.

Appendix A.5. Simulated Experiments

To simulate hypothetical data, the models described in Section Appendix A.1 and Section Appendix A.7 were used. That is, for a given sample size, I, true volumes, λ_i, were sampled for each participant, i, using a normal distribution, $N(0, \sigma_\lambda^2)$. For each participant and tool, k, mismeasurement of the volumes, were also sampled from a normal distribution, ϵ_{ik} $N(0, \sigma_\epsilon^2)$. Thus, the measurement for each participant's volume provided by each tool, denoted x_{ik}, was calculated as the sum of the true volume and the mismeasurement: $x_{ik} = \lambda_i + \epsilon_{ik}$. Intraclass correlations were calculated across these simulated measurements.

For each simulation, a target variable was generated for each participant, denoted $y_i k$, such that the target variable had a given correlation, ρ, with the true volumes in expectation

$$y_i = \rho \frac{\sigma_\delta}{\sigma_\lambda} \frac{\sigma_\lambda^2}{\sigma_\lambda^2 + \sigma_\epsilon^2} + \delta_i,$$

with δ_i representing variability in the target variable sampled from a normal distribution, $N(0, \sigma_\delta^2)$.

As described in the main text, simulations considered correlation values equal to 0.01, 0.1, and 0.2. Sample sizes were set to take values between 10 and 100 in steps of 10. For each combination of these parameters, simulated experiments were repeated 1,000,000 times (a high number of repetitions were needed to ensure the stability of estimates of the low rates). Simulated experiments with the UKB data were performed analogously to those with hypothetical data; instead of generating measurements by sampling true volumes and mismeasurement, the measurements sampled from the UKB dataset (UKB samples were chosen with replacement).

In all simulations with artificial data, several parameters described in Section Appendix A.1 do not influence results after setting the intraclass and interclass correlation and were therefore set to take arbitrary values: $\nu = 0$, $\sigma_\delta = 1$, and $\sigma_c = 0$. The remaining parameter, σ_λ was set to 0.019, a value estimated from the full UKB dataset using a linear mixed-effects model and restricted maximum likelihood as implemented by `lme4` (Bates et al., 2015).

To simulate hypothetical data, the models described in the previous sections were used. Sample sizes were set between 10 to 100 in steps of 10. At each combination of parameters, experiments were repeated 1,000,000 times. Simulated experiments with the UKB data were performed analogously to those with hypothetical data (UKB samples were taken with replacement).

Across repetitions, the rates of each effect were estimated by analytic Bayesian methods (binomial likelihood with Beta prior whose shape parameters were set to 1/2). For the effect of "Different Significance", the posterior was based on the number of simulations in which one correlation exhibited a p-value less than 0.05 and the other was above 0.05 (successes among relevant simulations) and the number of simulations in which at least one p-value was below 0.05 (total relevant simulations). The effect of "Different Direction" was calculated similarly, but used simulations in which both correlations had opposite magnitudes out of those in which both were significant. In the main text, ranges of uncertainty refer to 95% equal-tailed intervals and proportions refer to posterior medians.

The 95% equal-tailed interval of the posteriors for the simulations are shown in Figure A3.

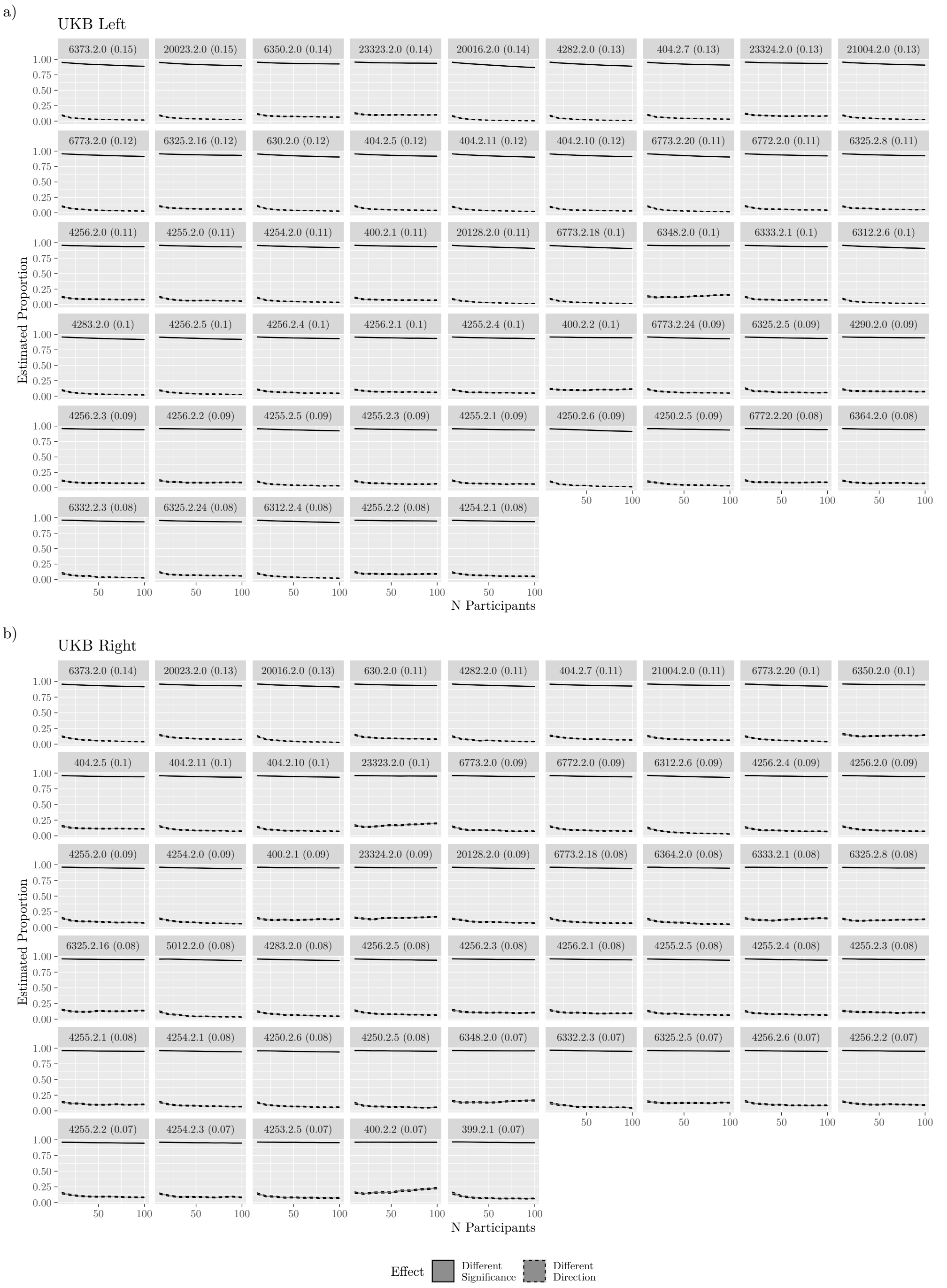

Figure A3: Effects of Low Measurement Consistency on the UKB in Left and Right Hemispheres. Ribbons span 95% equal-tailed interval estimated from simulated experiments. See also Figure 2, where the medians are represented with color.

Appendix A.6. Differing Significance Minimally Affected by Increasing Sample Sizes When ICC is Low

As described in the main text, when intraclass correlations were low, increasing the sample size affected the rates of differing significance only minimally Figure 2. This lack of influence can be understood by inspecting the distribution of simulated correlations Figure A4. When the intraclass correlation is low Figure A4, the significance of one correlation is nearly uninformative about the significance of the other (that is, the distributions of the two correlations are nearly circular). Moreover, the power to detect small correlations with even 100 participants is low, and so the power for two tests is very low. But when the intraclass correlation is higher Figure A4, the two correlations cluster, which improves the power of a second test conditioning on one test being significant.

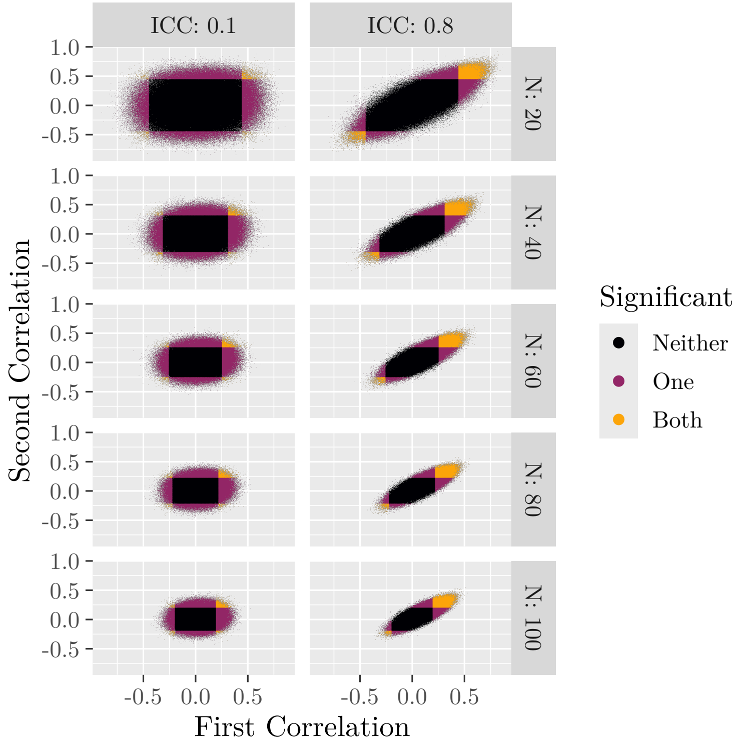

Figure A4: Correlations from Simulations with Artificial Data. Points correspond to simulations and are colored based on statistical significance. The figure shows only a subset of the simulated sample sizes (rows), only the smallest and largest intraclass correlations (columns), and only simulations in which the true effect size (correlation) was 0.1.

Appendix A.7. Measurement Error and Reduced Correlation Magnitude

Using the model from Appendix A.1, we can build a measurement noise model (e.g., Frost and Thompson, 2000). Call the final target for which we hope to find a relationship with the volume y. The relationship between that value and the volume was given by an ordinary linear regression with error δ.

$$y_i = \beta_0 + \beta_1 \lambda_i + \delta_i$$
$$\delta_i \sim N(0, \sigma_\delta^2)$$

But since we do not know λ, the volumes estimated by the tools are used instead, changing the regression coefficient as follows

$$y_i = \beta_0 + \tilde{\beta}_1 x_i + \delta_i$$

Dilution occurs because the coefficient $\tilde{\beta}_1$ estimated in this model tends to be closer to zero, decreased by a factor related to the intraclass correlation (Frost and Thompson, 2000).

$$\tilde{\beta}_1 = \beta_1 \frac{\sigma_\lambda^2}{\sigma_\lambda^2 + \sigma_\epsilon^2}$$

Correspondingly, the desired correlation, $\rho = cor(y, \lambda)$, will also be biased.

$$\beta_1 = \rho \frac{\sigma_\delta}{\sigma_\lambda}$$
$$\tilde{\beta}_1 = \tilde{\rho} \frac{\sigma_\delta}{sd(x)}$$
$$= \tilde{\rho} \frac{\sigma_\delta}{\sqrt{\sigma_\epsilon^2 + \sigma_\lambda^2}}$$
$$\Longrightarrow$$
$$\rho \frac{\sigma_\delta}{\sigma_\lambda} = \tilde{\rho} \frac{\sigma_\delta}{\sqrt{\sigma_\epsilon^2 + \sigma_\lambda^2}} \frac{\sigma_\lambda^2 + \sigma_\epsilon^2}{\sigma_\lambda^2}$$
$$\Longrightarrow$$
$$\rho = \tilde{\rho} \frac{\sqrt{\sigma_\epsilon^2 + \sigma_\lambda^2}}{\sigma_\lambda}$$

Appendix A.8. Correlations Between Volumes of the Amygdala and Cognitive Variables

As described in the main text, a set of 50 “cognitive” variables from the UKB was selected for each hemisphere. Selection was based on the rank correlation between the variable and the average (across methods) amygdalar volume. The correlations between those variables and the original volume estimates are shown in Figure A5. For both hemispheres, the magnitude of the correlations with the volumes as reported by FreeSurfer tended to be higher than those reported by FSL.

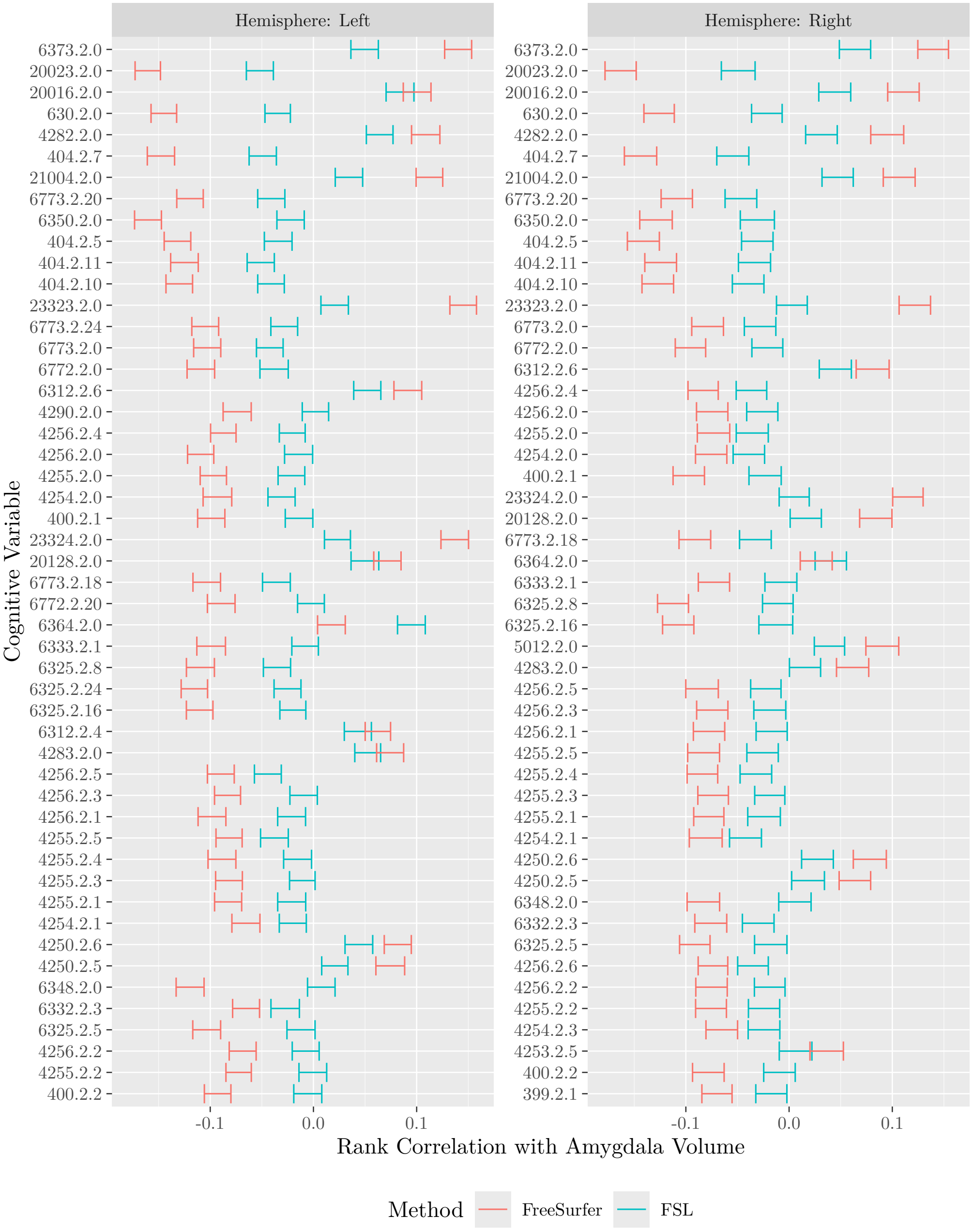

Figure A5: Correlations Between Cognitive Factors and Estimated Volumes of the Left and Right Amygdala. Variables are ordered by decreasing rank correlation using average of left hemisphere volume estimates. Note that variables were selected based on the magnitude of their correlation with amygdalar volumes, which differed across hemispheres, and so the variables in left and right panels differ. Error bars span 95% confidence intervals (bootstrapped with 1000 samples).
